# Insights into the function of HDAC3 and NCoR1/NCoR2 co-repressor complex in metabolic diseases

**DOI:** 10.3389/fmolb.2023.1190094

**Published:** 2023-08-22

**Authors:** Harikrishnareddy Paluvai, Kumar D. Shanmukha, Jens Tyedmers, Johannes Backs

**Affiliations:** ^1^ Institute of Experimental Cardiology, Heidelberg University, Heidelberg, Germany; ^2^ DZHK (German Center for Cardiovascular Research), Partner Site Heidelberg/Mannheim, Heidelberg, Germany

**Keywords:** HDAC3, NCoR1, SMRT, GPS2, PPARs

## Abstract

Histone deacetylase 3 (HDAC3) and nuclear receptor co-repressor (NCoR1/2) are epigenetic regulators that play a key role in gene expression and metabolism. HDAC3 is a class I histone deacetylase that functions as a transcriptional co-repressor, modulating gene expression by removing acetyl groups from histones and non-histone proteins. NCoR1, on the other hand, is a transcriptional co-repressor that interacts with nuclear hormone receptors, including peroxisome proliferator-activated receptor gamma (PPARγ) and liver X receptor (LXR), to regulate metabolic gene expression. Recent research has revealed a functional link between HDAC3 and NCoR1 in the regulation of metabolic gene expression. Genetic deletion of HDAC3 in mouse models has been shown to improve glucose intolerance and insulin sensitivity in the liver, skeletal muscle, and adipose tissue. Similarly, genetic deletion of NCoR1 has improved insulin resistance and reduced adiposity in mouse models. Dysregulation of this interaction has been associated with the development of cardio-metabolic diseases such as cardiovascular diseases, obesity and type 2 diabetes, suggesting that targeting this pathway may hold promise for the development of novel therapeutic interventions. In this review, we summarize the current understanding of individual functions of HDAC3 and NCoR1/2 and the co-repressor complex formation (HDAC3/NCoR1/2) in different metabolic tissues. Further studies are needed to thoroughly understand the mechanisms through which HDAC3, and NCoR1/2 govern metabolic processes and the implications for treating metabolic diseases.

## 1 Overview of HDACs and co-repressor complex

Histones are unique proteins that regulate transcription by aiding DNA condensation into nucleosomes. An octamer of histones is composed of eight subunits, two each of H2A, H2B, H3, and H4. Histone acetylation and methylation regulates the tightness of DNA wrapping (Bilokapic et al., 2018; Lawlor and Yang, 2019). Histone deacetylases (HDACs) are the enzymes responsible for removing the acetyl group from histone proteins, thus promoting the DNA’s tight wrapping, making it less accessible to transcription factors, thereby leading to gene repression (Lawlor and Yang, 2019). Deacetylation of histones influences transcription, whereas deacetylation of non-histone proteins promotes protein stability, interactions, and signaling (Kim and Bae, 2011). Post-translational acetylation of histone and other non-histone proteins is determined by the equilibrium between acetylation and deacetylation (Wang et al., 2012).

**FIGURE 1:**
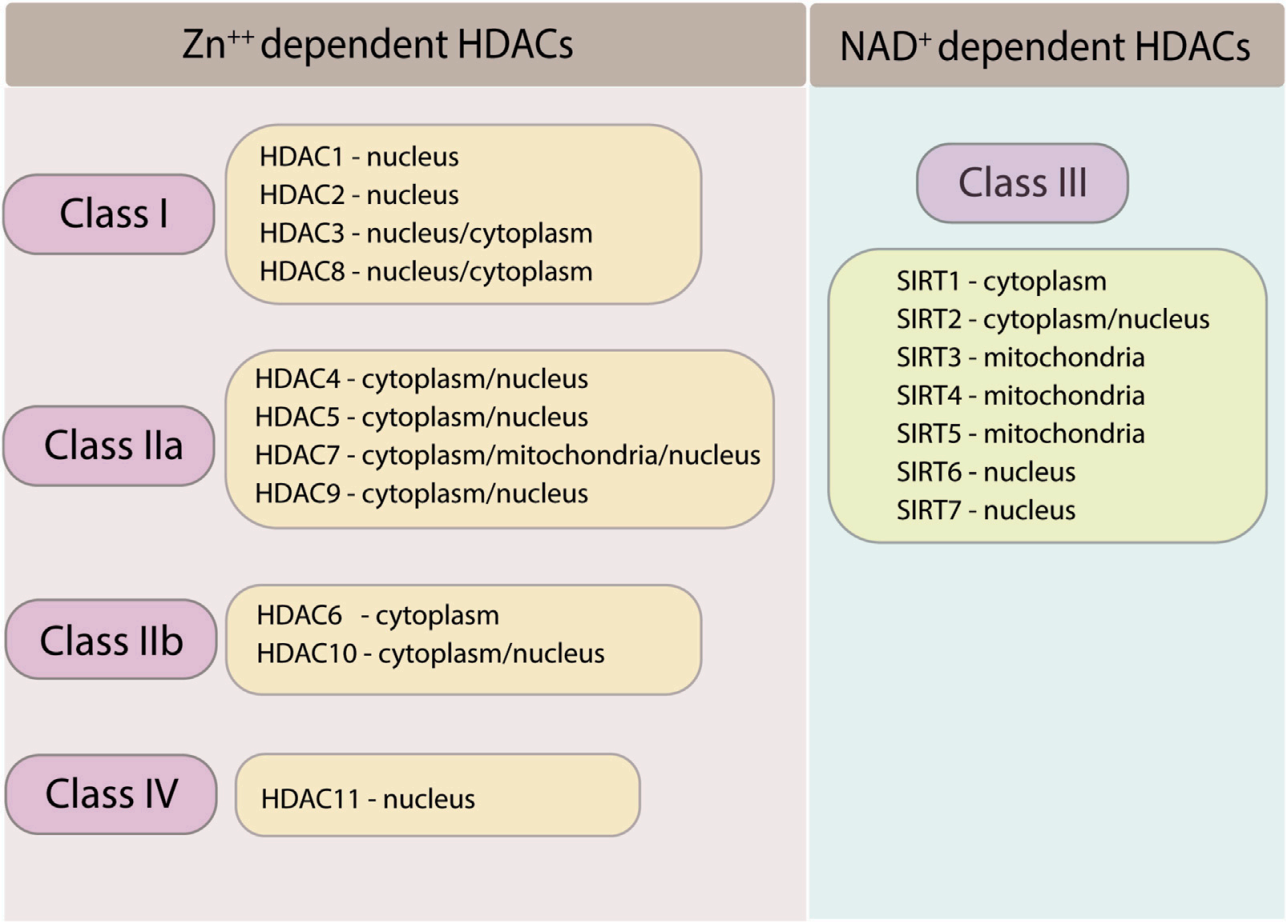
Classification of HDACs family and their sub cellular localization.

To date, 18 HDACs have been identified in mammals based on homology to yeast deacetylases. According to their sequence identity and catalytic activity, they are divided into four classes (Yang and Seto, 2008; Shakespear et al., 2011) (refer to Figure 1). The Rpd3/Hda1 family includes zinc-dependent HDACs of classes I, II, and IV, while the NAD + -dependent sirtuin family is classified as class III HDACs (Ruijter et al., 2003). HDAC1, 2, 3, and 8 all belong to HDAC Class I. Class II HDACs are further classified into class IIa (HDAC4, 5, 7, and 9) and class IIb (HDAC6 and 10). Class III HDACs include SIRT1-7, while class IV HDACs include HDAC11 (Ruijter et al., 2003; Gregoretti et al., 2004). Class I HDACs have a nuclear localization signal (NLS), but they usually do not possess a nuclear export signal (NES). The only exception to this rule is histone deacetylase 3 (HDAC3), which has both a NLS and a NES (Wang et al., 2012). As a result, class I HDACs primarily reside in the nucleus and regulate the expression of numerous genes, including thioredoxin binding protein-2 (TBP-2), myocyte enhancer factor-2 (MEF2), Nuclear factor kappa B (NFkB), and GATA (Kato et al., 2009; Wang et al., 2012). The formation of multiprotein complexes is the fundamental principle by which class I HDACs carry out their functions (Xiong et al., 2020). They interact with several large complexes, including the co-repressing RE1 silencing transcription factor/neural restrictive silencing factor (CoREST), nucleosome remodeling and deacetylase (NuRD), nuclear receptor co-repressors (NCoRs) NCoR1 and NCoR2 [also known as silencing mediator of retinoid and thyroid receptors (SMRT)], and switch-independent 3A (Sin3A) complexes. Interestingly, HDAC3 purified recombinant protein does not have intrinsic enzymatic activity and depends on the deacetylase activation domain (DAD) of either NCoR1 or NCoR2 or both for its activity (Guenther et al., 2001a). Any mutations on DAD affect HDAC3 protein stability and enzymatic activity (Sun et al., 2013). Furthermore, HDAC3 is well known to interact with class IIa HDACs (HDAC4, 5, and 7) (Fischle et al., 2002). In vertebrates, class IIa HDACs have poor enzymatic function on acetylated lysines due to tyrosine/histidine substitution in the catalytic region (Lahm et al., 2007; Di Giorgio et al., 2021). Hence, class IIa HDACs rely on their association with HDAC3 and NCoR1/NCoR2 complex to mediate transcriptional repression of the target genes (Fischle et al., 2002; Di Giorgio et al., 2015; Di Giorgio and Brancolini, 2016; Paluvai et al., 2020). Therefore, HDAC3 is the critical enzyme that confers deacetylase enzymatic activity by interacting with NCoR1/NCoR2 complex and class IIa HDACs. Furthermore, specific mutations on the deacetylase domain of class IIa HDACs suppress the enzymatic activity and result in loss of HDAC3 binding (Fischle et al., 2002).

**FIGURE 2:**
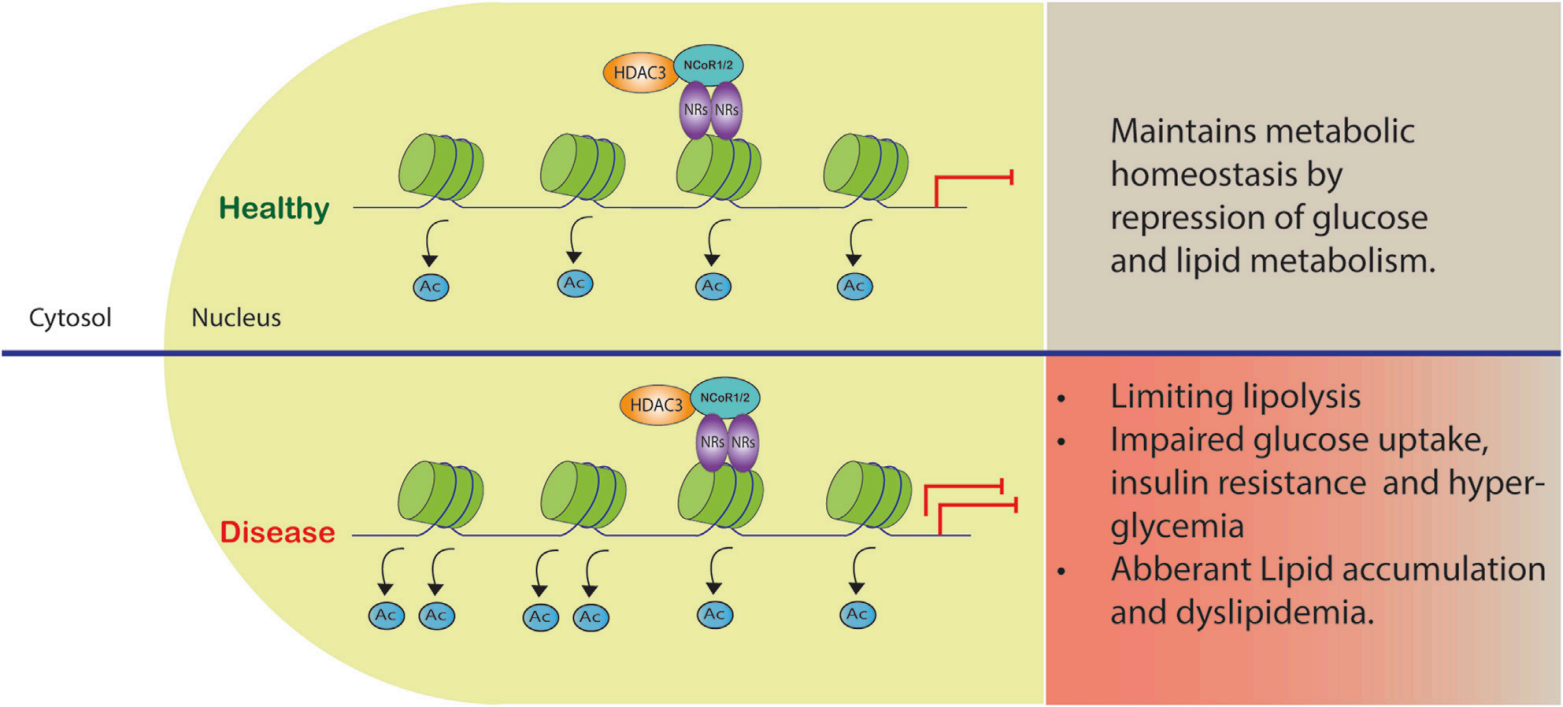
Co-repressor complex dysregulation in metabolic diseases. The complex formed by HDAC3 and NCoR1/2 reinforces gene repression by acting as a co-repressor. Certain gene promoters are recruited to this complex, leading to reduced gene transcription and metabolic dysfunction.

Genetic deletion of NCoR1 during development leads to embryonic lethality resulting in hematologic and neurologic abnormalities, while genetic deletion of NCoR2 causes embryonic lethality due to cardiac ventricular development defects (Jepsen et al., 2000; Jepsen et al., 2008). Importantly, NCoR1 and NCoR2 mediate at least a part of their biological functions through the recruitment and activation of HDAC3. Several nuclear receptors (NR) are involved in the regulation of mitochondrial biogenesis. The PPAR family has been found to be essential for oxidative metabolic regulation in a variety of tissues. PPARα, in particular, increases the expression of genes involved in mitochondrial β-oxidation (Gulick et al., 1994). PPARγ promotes mitochondrial biogenesis in white adipose tissue (WAT) (Wilson-Fritch et al., 2004) and induces mitochondrial genes in brown adipose tissue for thermogenic program (Puigserver et al., 1998). HDAC3 and NCoR complex represses majority of the NRs involved in mitochondrial biogenesis. For example, liver-specific NCoR1 deletion increased transcription of mitochondrial oxidative phosphorylation (OxPhos) genes and genes related to PPARα and Estrogen-related receptor alpha (ERRα) signaling pathways, indicating the critical role of NCoR1 derepression in PPARα/ERRα transactivation (Jo et al., 2015). A similar pattern was also observed in WAT mice lacking HDAC3 (Ferrari et al., 2017). Deletion of HDAC3 also has a lethal effect during development (Montgomery et al., 2008; Bhaskara et al., 2008). Epidermal-specific HDAC3-KO mice exhibit a similar phenotype to that seen in NCoR1 and NCoR2 KO during embryogenesis in the epidermis, leading to mortality due to a compromised external defense barrier (Szigety et al., 2020). In liver specific HDAC3-KO mice, both NCoR1 and NCoR2 have identical roles in regulating lipid metabolism (Sun et al., 2012; Feng et al., 2011). These results suggest that HDAC3 and nuclear receptor co-repressors have similar functions and repress the same set of genes *in vivo* which would be in line with the notion that they act in a complex. Dysregulation of this HDAC3 and NCoR1/2 complex can contribute to the development of metabolic syndrome, a cluster of interconnected metabolic abnormalities, including insulin resistance, dyslipidemia, and obesity (Figure 2). In this review, we summarize the physiological role of HDAC3 and co-repressor complex NCoR1/NCoR2 in different metabolic tissues like adipocytes, liver, heart, and skeletal muscles.

## 2 Adipocytes

Adipose tissue is crucial for systemic glucose and lipid homeostasis (Rosen and Spiegelman, 2014). Its principal function is to store excess energy as triglycerides and hydrolyze triglycerides during periods of dietary restriction. Previous findings indicate that adipocytes may also function as endocrine organ that produce lipid metabolites, hormones, and chemokines to coordinate systemic glucose and lipid metabolism (Waki and Tontonoz, 2007). Thus, both excessive and insufficient adipogenesis are directly linked to the dysregulation of energy metabolism, including obesity, type 2 diabetes, and lipodystrophy. There are two types of adipose tissue: white (WAT) and brown (BAT) (Giralt and Villarroya, 2013). BAT arises during embryonic development and is primarily responsible for heat production due to the activity of uncoupling protein 1 (UCP1), which decouples the inner mitochondrial membrane respiratory chain from ATP synthesis (Sidossis and Kajimura, 2015). In contrast, WAT is essential for energy storage in the form of triacylglycerols (Kurylowicz and Puzianowska-Kuznicka, 2020). BAT and WAT also differ significantly in their morphology and function. In response to specific environmental cues (such as exposure to cold), WAT shifts to a brown-like phenotype in a process known as browning (Fisher et al., 2012; Ohno et al., 2012).

PPARγ is a key factor controlling the function of adipose tissue in whole-body glucose metabolism (Evans et al., 2004; Lehrke and Lazar, 2005; Tontonoz and Spiegelman, 2008). Nuclear receptors like PPARγ (Tontonoz and Spiegelman, 2008) are significantly abundant in adipose tissue and play an essential role in adipocyte differentiation, insulin sensitivity, and adipokine/cytokine secretion. NR regulate gene expression by recruiting co-activators or co-repressor complexes to alter adjacent histone post-translational modifications (Glass and Rosenfeld, 2000). These co-regulators are promising targets for therapy of a variety of metabolic diseases (Lonard and O'Malley, 2012). One such co-repressor complex is the nuclear receptor co-repressor (NCoR1/NCoR2/HDAC3) complex (Lonard and O'Malley, 2012; Guenther et al., 2001a; Fowler and Alarid, 2004; Feige and Auwerx, 2007).

Previous research demonstrated that deletion of NCoR1 in adipocytes increases insulin sensitivity, decreases inflammatory responses in mice and prevented substantial weight gain induced by high-fat diet (Li et al., 2011). The phenotype of these animals resembled that of mice treated with thiazolidinediode (TZD), a potent PPARγ agonist. Previous research has shown that Cyclin-dependent kinase 5 (CDK5) phosphorylates PPARγ, reducing its function and contributing to insulin resistance (Choi et al., 2010). Depletion of NCoR1 in WAT increases the amount of insulin-sensitizing, unphosphorylated PPARγ, implying that NCoR1 promotes PPARγ phosphorylation via CDK5 (Li et al., 2011). Global deletion of NCoR2 in adult mice induces visceral obesity independent of a high-fat diet or increased food intake (Shimizu et al., 2019).

Alessandra et al. demonstrated that deletion of HDAC3 in adipose tissue (H3atKO) enhanced browning of white fat and promoted global thermogenesis without changing BAT phenotype (Ferrari et al., 2017). Another genetic model showed that HDAC3 is required for thermogenesis through deacetylating and activating peroxisome proliferator-activated receptor gamma co-activator 1-alpha (PGC-1α), a co-activator for thermogenic ERRα (Emmett et al., 2017). Genetic deletion of HDAC3 in BAT affected thermogenesis, and the mice died from acute cold exposure. Moreover, UCP1 is nearly absent in these mice, and mitochondrial oxidative phosphorylation (OXPHOS) genes are downregulated, thereby limiting mitochondrial respiration (Emmett et al., 2017).

Surprisingly adipocyte co-deletion of NCoR1/NCoR2 in both male and female mice resulted in hypoglycemia, hypothermia, and a fatal phenotype occurred within 10 days (Ritter et al., 2021). Moreover, HDAC3 protein expression is greatly reduced in these mice, and the mice showed less HDAC3 enzymatic activity. This behavior was not observed in animals lacking either NCoR1 or NCoR2 alone. Furthermore, when HDAC3 was deleted, all mice survived despite moderate weight loss, hypoglycemia, and less adipose tissue (Ritter et al., 2021). HDAC3 enzymatic activity depends on interaction with NCoR1/NCoR2 (Sun et al., 2013). Therefore, genetic loss of NCoR1/2 in BAT (NCoR1/2 BAT-dKO) leads to loss of HDAC3 enzymatic activity. Correspondingly, NCoR1/2 BAT-dKO mice have in common with HDAC3 BAT-KO mice a dysregulation of BAT lipid metabolism (Richter et al., 2022). Despite these similarities, deletion of NCoR1/2 in BAT does not phenocopy the cold sensitivity observed in HDAC3 BAT-KO. In contrast, BAT deficient for NCoR1/2 displays inflammation, especially with regard to the interleukin-17 axis, which promotes thermogenic capacity by increasing innervation (Richter et al., 2022).

Genetic disruption of the interaction of NCoR and HDAC3 [referred to as DADm, with a single amino acid substitution (Y478A) in the NCoR1 DAD] resulted in decreased body weight and whole-body fat as well as loss of co-repressor activity of NCoR1 and HDAC3 enzymatic activity (Alenghat et al., 2008). DADm mice were resistant to diet-induced obesity and protected from insulin resistance when fed a high-fat diet (HFD) (Alenghat et al., 2008). Fang et al. (2011) reported that disruption of the interaction of nuclear receptors to the receptor interaction domain (RID) of NCoR2 in a mouse model (referred to as SMRT^mRID1^) led to massive lipid accumulation in white and brown adipose tissue, and the mice developed multiple metabolic dysfunctions such as insulin resistance and excessive weight gain when provided with a high-fat diet. The hypertrophic phenotype of SMRT^mRID1^ mice is associated with altered mitochondrial activity, which is likely caused by a reduction in mitochondrial biogenesis and fatty acid oxidation. The lower mitochondrial activity in SMRT^mRID1^ mice could be attributed to decreased expression of PGC1α, a major modulator of mitochondrial biogenesis and activity (Fang et al., 2011). HDAC3 and NCoR1/2 corepressor complex also interact with other subunits in adipocytes to control glucose homeostasis and insulin resistance. G protein pathway suppressor 2 (GPS2), a subunit of the NCoR2 and HDAC3 complex, plays a crucial role in the adipocyte functions in the context of obesity and type 2 diabetes (Treuter et al., 2017; Cardamone et al., 2018; Drareni et al., 2018). One study showed that adipocyte-specific GPS2 (GPS2 AKO) deficient mice exhibited increased adiposity (fat accumulation), impaired glucose tolerance, and insulin resistance (Drareni et al., 2020) similar to SMRT^mRID1^ mutant model (Fang et al., 2011). HDAC3/NCoR2/GPS2 directly inhibit HIF-1α transcriptional activity. HIF-1α has been shown to interfere with PGC-1α function in adipocytes, causing changes in adipocyte fatty acid catabolism (Krishnan et al., 2012). It is also worth noting that corepressor complex, has been shown to deacetylate PGC-1α in brown adipocytes (Emmett et al., 2017) and loss of HDAC3 in adipocytes caused deficient WAT remodeling and browning (Ferrari et al., 2017). Interestingly, Basse et al. (2017) reported that WAT browning was severely hindered in GPS2 AKO after cold exposure or β3-adrenergic receptor activation, implying that GPS2 interact with HDAC3 to influence PGC-1α-dependent browning in addition to HIF-1α. These results clearly demonstrate that HDAC3 and NCoR1 generally function as adipogenesis negative regulators, controlling the development of preadipocytes into mature adipocytes. Dysregulation of these enzymes can result in increased adipocyte differentiation, which can lead to increased adipose tissue expansion and perhaps contribute to obesity. Increased HDAC3 or NCoR1 activity could limit lipolysis, resulting in a reduction in the breakdown of stored triglycerides and perhaps contributing to insulin resistance. HDAC3 inhibition or disruption of the HDAC3-NCoR1 corepressor complex has been shown to improve insulin sensitivity by increasing lipolysis in adipocytes thereby reducing adipose tissue mass. This suggests that HDAC3 and NCoR1 play important roles in the regulation of lipid metabolism in adipose tissue. However, transgenic mice models that overexpress HDAC3 or NCoR1 in white adipose tissue can also provide molecular insights into their regulatory activities. This approach could provide valuable information in better understanding the effects of increased HDAC3 or NCoR1 activity on adipocyte function and their impact on whole-body metabolism.

Overall, studies on HDAC3 and NCoR1 in adipocytes highlight their involvement in adipocyte differentiation, adipose tissue expansion, and lipid metabolism. Targeting these factors may hold therapeutic potential for metabolic disorders such as obesity and insulin resistance. However, further research is needed to fully elucidate the underlying molecular mechanisms and to explore the specific therapeutic implications of manipulating HDAC3 and NCoR1 in adipose tissue.

## 3 Liver

**FIGURE 3:**
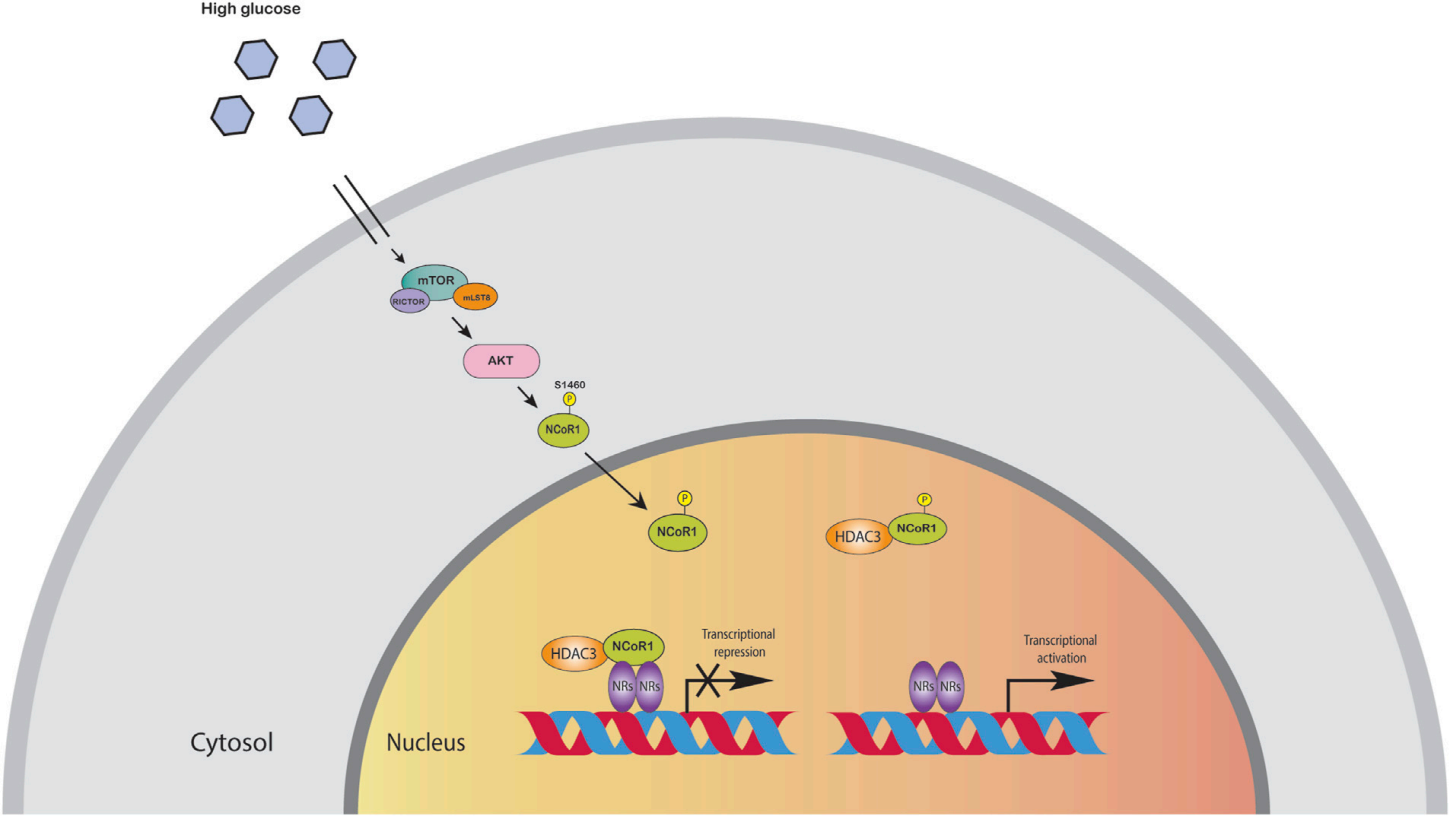
High amounts of glucose and insulin phosphorylate NCoR1 by AKT. Phosphorylation of NCoR1 changes its activity from co-repressor to co-activator, thereby activating NRs.

The liver is the major organ for glucose and lipid metabolism. Lipids stored in the liver originate from three primary sources: free fatty acids in the blood, *de novo* lipogenesis, and food intake. HFD induced metabolic pathways can promote oxidative stress in mitochondria and the endoplasmic reticulum, as well as *de novo* lipogenesis and inflammation. Moreover, these metabolic processes are controlled by transcription factors and co-regulator networks (Mouchiroud et al., 2014; Musso et al., 2016). Several liver-focused studies suggest that NCoR, together with HDAC3 corepressor complex, has a dominant physiological role in hepatocytes (Liang et al., 2019a). Disruption of these corepressor networks by genetic, environmental, or dietary variables can result in metabolic dysregulation, which has been linked to the progression of non-alcoholic fatty liver disease (NAFLD) to steatohepatitis and even liver cancer (Liang et al., 2019a). Liver specific knockout (LKO) of NCoR replicated the metabolic changes reported in HDAC3-depleted livers, including hepatic lipid accumulation, reciprocal glycogen decrease, and upregulation of hepatic lipogenesis. The transcriptome profiling of NCoR1 and HDAC3 KO livers revealed similarities, as the upregulated genes were heavily enriched in lipid and fatty acid metabolism, consistent with the lipid metabolic abnormalities (Knutson et al., 2008; Feng et al., 2011; Sun et al., 2012; Shimizu et al., 2015; Zhang et al., 2018a; Liang et al., 2019a; Liang et al., 2019b). During the fasting-to-feeding transition, the role of NCoR1 in liver energy metabolism is particularly intriguing: both HDAC3 and NCoR1 are known to repress genes involved in lipogenesis, but paradoxically, NCoR1 has also been reported to be essential for inhibition of PPARα, hepatic fatty oxidation, and ketogenesis (Alenghat et al., 2008; Sun et al., 2013). NCoR1 can select its repressor targets dependent on the energy state of the cell to regulate energy metabolism (Jo et al., 2015). High amounts of glucose and insulin during feeding activate the target of rapamycin complex 1 (mTORC1)-AKT signaling cascade, thereby phosphorylating serine 1460 (pS1460) of NCoR1 (pS1460 NCOR1). Phosphorylation of NCoR1 at serine 1460 inhibits its repressive interaction with LXR, thereby activating lipogenic LXR target genes (Jo et al., 2015; Jo et al., 2015). In contrast, it facilitates NCoR1’s interaction with PPARα and ERRα, making it more difficult for ketogenic and Mitochondrial Oxidative Phosphorylation (OXPHOS) genes to be activated (Jo et al., 2015). More interestingly, pS1460 NCoR1 also showed reduced HDAC3 enzymatic activity indicating unphosphorylated form of NCoR1, acts as a co-repressor that represses gene expression by recruiting HDACs to gene promoters (Figure 3) (Jo et al., 2015). Phosphorylation of NCoR1 changes its activity from co-repressor to co-activator while promoting the recruitment of histone acetyltransferase (HATs) and resulting in a feedback loop in which a better metabolic phenotype is observed in the mice. This pathway has the potential to be a therapeutic target for reversing the metabolic diseases such as insulin resistance and dyslipidemia. Thyroid hormone controls serum lipids and enhances hepatic fatty oxidation (Song et al., 2011). In addition to promoting mitochondrial uncoupling and biogenesis, thyroid hormone also influences glucose metabolism, as low levels or resistance to thyroid hormone correlate with insulin resistance. The liver primarily mediates these effects of thyroid hormone on systemic lipid and glucose metabolism, but in adipose tissue, it decreases lipid storage and increases fatty acid oxidation. Vella and Hollenberg (2017), generated a mouse model lacking two key nuclear receptor interacting domains (RID) NCoRδRID in the liver to examine the role of NCoR1 in thyroid hormone receptor (TR) modulation. Consequently, they cannot interact with TRs or LXRs (Astapova et al., 2008). Intriguingly, they observed that mice with a defective nuclear receptor binding region have lower cholesterol levels in the liver and a higher alternative bile acid production. The enhanced expression of alternative bile acid production genes results from de-repression of TRβ1 in the mutant NCoR1 mice (Astapova et al., 2014). Additional studies showed that hepatocyte proliferation was also increased in NCoRδRID animals, suggesting that NCoR1 may have a role in regulating cell proliferation (Feng et al., 2001).

GPS2, a subunit of the NCoR and HDAC3 complex, plays a crucial role in the progression of non-alcoholic steatohepatitis (NASH) (Liang et al., 2019b). GPS2 protects the liver by interacting with sumoylated nuclear receptors such as LRH-1 and LXRβ to inhibit the expression of inflammatory cytokines during the acute phase response of the liver. Liver-specific GPS2 knockout mice are protected from diet-induced steatosis and fibrosis and activation of PPARα driven lipid catabolic genes (Liang et al., 2019b). Derepression of PPARα in this mouse seems to depend on NCoR1 and HDAC3 but not on NCoR2. The enzymatic activity of HDAC3 requires NCoR in the liver (Sun et al., 2013). HDAC3 deletion in the liver resulted in severe hepatic steatosis and elevated expression of lipogenic enzymes, yet these mice are sensitive to insulin, perhaps owing to the limited availability of gluconeogenic genes (Knutson et al., 2008; Feng et al., 2011; Sun et al., 2013). Other research conducted by Bochkis et al. on aged mice revealed that HDAC3 was colocalizing with PPARα (including PPARγ and LXRα) in promoters of a subset of lipogenesis genes that resulted in hepatosteatosis (Bochkis et al., 2014), and it is striking that the phenotype found in these mice is comparable to that of HDAC3-KO in the liver (Bochkis et al., 2014). In addition, this study demonstrated that NCoR1 is repressed in aged livers resulting in inactivation of HDAC3 and activation of nuclear receptors (Bochkis et al., 2014). In agreement with the above-mentioned findings, previous studies on human liver HepG2 cells showed that treatment with sodium butyrate, a class I HDAC inhibitor (that primarily inhibits HDAC1, 2, and 3), increased the production of Fibroblast growth factor 21 (FGF21). This increase was associated with a disruption of the interaction between PPARα and HDAC3 at the FGF21 promoter level (Li et al., 2012). FGF21 increased fatty acid oxidation in the liver and ameliorates metabolic dysregulation in obese individuals (Xu et al., 2009). However, the HDAC3-KO in liver had no increase in FGF21 expression and the mice showed severe hepatosteatosis (Feng et al., 2011). Later, it was found that the main targets of HDAC3 in the liver were the nuclear receptors RevErbs. RevErb brings in NCoR and HDAC3 to suppress target gene expression, and under physiological conditions, this function likely overwrites the effects of PPARα. Indeed, liver KO of NCoR and RevErb mice showed liver steatosis phenotype, resembling that of the liver HDAC3-KO mice (Astapova et al., 2008; Cho et al., 2012).

NCoR1-DADm mice, when fed with high-fat diet, were resistant to obesity and developed insulin resistance while having decreased hepatic glucose production (Alenghat et al., 2008). This finding was unexpected since Rev-Erbα normally works to suppress hepatic glucose synthesis by recruiting NCoR1-HDAC3 to gluconeogenic genes. More interestingly, the DADm mice showed no signs of hepatic steatosis in contrast to the HDAC3-KO mice, and the authors claimed that the phenotype is independent of HDAC3 deacetylase activity. This may also indicate a protective impact of residual NCoR2-dependent HDAC3 activity (Alenghat et al., 2008).

A study showed that the autophagy-lysosomal pathway regulates the stability of NCoR1 and HDAC3 co-repressor complex in hepatocytes. Under fasting conditions, both HDAC3 and NCoR1 are degraded, and autophagy-deficient (Vps15) mice impaired the degradation of this complex and inhibited hepatic PPARα activity, resulting in lipid oxidation (Iershov et al., 2019). Notably, the pharmacological inhibition of HDACs and administration of a PPARα synthetic ligand were sufficient to markedly enhance mitochondrial function in Vps15-deficient livers (Iershov et al., 2019).

Mutations in the X-linked gene encoding methyl-CpG-binding protein 2 (MECP2) directly regulate lipid metabolism via interacting with NCoR1/NCoR2 and recruiting HDAC3 to target genes, to remove the histone modifications in the liver (Kyle et al., 2016). MECP2 deletion in the liver resulted in fatty liver disease and dyslipidemia comparable to the phenotype generated by HDAC3 liver-specific deletion. In addition, deletion of MECP2 in the liver dramatically impairs HDAC3 capacity to bind regulatory regions surrounding the transcription start site (TSS) of squalene monooxygenase (SQLE), fatty acid transporter (CD36), and Fatty acid synthase (FASN) (Kyle et al., 2016). Taken together, HDAC3 and NCoR1 KO in the liver contributes to metabolic imbalances, resulting in hepatic steatosis, insulin resistance, altered glucose and lipid metabolism. HDAC3 KO phenotype can be rescued by wild-type or catalytically inactive HDAC3 mutants. Since NCoR1 DADm mice improved the metabolic phenotype, it would be interesting to see if NCoR1 DAD mutant mice can rescue the metabolic phenotype in NCoR1 LKO. Understanding the specific mechanisms by which HDAC3 and NCoR1 interact with lipid metabolism genes is essential for unraveling their roles in liver function and exploring potential therapeutic targets for metabolic diseases related to lipid metabolism.

## 4 Skeletal muscle

Skeletal muscle is the metabolically most active organ in the human body, accounting for approximately 18% of total daily energy expenditure (Scheja and Heeren, 2019). Skeletal muscle is regarded as an endocrine and paracrine organ. It secretes Interleukins (IL-6 and IL-7) and interacts with numerous tissues and organs (like liver, adipose tissue, pancreas, bone marrow, and the cardiovascular system) (Pedersen and Febbraio, 2012; Mizgier et al., 2019). Muscular contraction is one of the key physiological functions of skeletal muscle and helps to maintain organ and systemic metabolic balance (Guo et al., 2020). Skeletal muscle remodeling is tightly controlled by many transcription factors and co-regulator/co-repressor complexes that can affect chromatin structure and regulate gene transcription (Kouzarides, 2007; Santos et al., 2011).

Yamamoto et al. (2011) found that skeletal muscle specific deletion of NCoR1 (NCoR1-SMKO) in mice resulted in a normal phenotype on a standard chow diet. However, when the mice were subjected to high fat diet, their muscle fiber size and exercise endurance increased, indicating that NCoR1 plays a suppressive role in muscle reprogramming. Furthermore, the NCoR1 SMKO mice on high fat diet exhibited increased oxidative muscle metabolism and mitochondrial abundance. These findings are consistent with known phenotypes associated with inhibition of nuclear receptors involved in this process, such as PPARδ, by NCoR1, and with the suppression of MEF2, which NCoR1/HDAC3 may coordinately inhibit. In addition, MEF2-dependent genes were elevated in NCoR1-SMKO mice. This can be explained by enhanced acetylation of histones in target genes and activity of the MEF2D transcription factor due to the instability of the NCoR1/NCoR2/HDAC3 complex. NCoR1 activity appeared to be dynamically regulated in the muscle, since its expression and/or nuclear localization were reduced in circumstances where fatty acid oxidation was stimulated, such as in long-term fasting, high-fat eating, and endurance exercise (Yamamoto et al., 2011). Given that HDAC3 interacts with NCoR1 predominantly in the nucleus, it is intriguing to observe the expression and activity of HDAC3 under these settings. HDAC3-SMKO mice showed glucose intolerance and muscle insulin resistance, but surprisingly, these mice showed enhanced exercise capacity and muscle fatigue resistance compared to wild-type (WT) littermate controls (Hong et al., 2017). HDAC3-SMKO might promote a shift towards more fatigue-resistant slow-twitch fibres, which are better suited for endurance activities. Furthermore, HDAC3 deficiency may result in improved calcium handling in muscle cells, which may contribute to enhanced muscular function and fatigue resistance in the mice. Moreover, in these mice, insulin signaling cascades such as pAKT/AKT, pIRS1-S1101, and pGSK/GSK are unaffected; however, glucose uptake and insulin sensitivity are reduced in the glucose tolerance test (GTT) and insulin tolerance test (ITT) (Hong et al., 2017). It would be interesting to see if changes in fibre type distribution, such as a shift towards fast-twitch fibres, contribute to insulin resistance in HDAC3-SMKO. Additional studies show that HDAC3 deletion increases the expression of the first rate-limiting enzyme in purine metabolism in skeletal muscles, adenosine monophosphate deaminase 3 (AMPD3) (Hong et al., 2017). AMPD3 can deaminate AMP to produce IMP (inosine monophosphate), accelerating the conversion of aspartic acid to fumarate and malate in the tricarboxylic acid cycle (TCA cycle) (Hong et al., 2017). Furthermore, it was observed that the glycolysis flux rate in HDAC3-SMKO mice was reduced in response to exercise-induced glucose tagged ^13^C_6_ but higher in TCA cycle intermediates (Hong et al., 2017; Gong et al., 2018). This is also true in the case of HDAC3 deacetylase dead mutant (NS-DADm) global knock-in mice, where glucose uptake was significantly lower during the exercise (Song et al., 2019). RNA-seq analysis of NS-DADm skeletal muscles displayed a similar gene expression pattern as compared to HDAC3-SMKO. As a result, mitochondrial oxidation is activated by a general rise in TCA cycle metabolites. This might also explain why MCH3-HDAC3 KO (postnatal deletion of HDAC3 in cardiac and skeletal muscle) animals die prematurely when exposed to a high-fat diet. MCH3-HDAC3 KO mice display severe hypertrophic cardiomyopathy/fibrosis and lipid accumulation in the heart and skeletal muscle (Sun et al., 2011). Knockout of HDAC3 in the liver results in severe hepatosteatosis, which can be rescued by wild-type or catalytically inactive mutants of HDAC3 (but this mutant still binds to NCoR1), demonstrating a role for HDAC3 that is independent of its enzymatic activity *in vivo* (Sun et al., 2013). However, enzymatically inactive mutant HDAC3 (NS-DADm) mice cannot recover the muscle fuel switching seen in muscle-specific HDAC3-KO (mKO) mice (Song et al., 2019). These studies show that HDAC3 enzymatic activity is indispensable for the metabolic function in the skeletal muscle. Moreover, global disruption of the NCoR1-HDAC3 complex (DADm) in mice improved energy metabolism (e.g., insulin sensitivity and oxygen consumption) in skeletal muscle (Alenghat et al., 2008). Additional research demonstrated that MECP2 liver-specific KO mice exhibited glucose intolerance and insulin resistance in skeletal muscle, as seen by impaired glucose uptake and shift to fatty acid oxidation for energy production (Kyle et al., 2016). Overall, HDAC3 and NCoR1 play important roles in the regulation of metabolic pathways in skeletal muscle. Absence of these co-repressors may lead to alterations in the expression of genes involved in glucose and lipid metabolism, leading to metabolic dysfunction. This can contribute to insulin resistance, impaired glucose uptake, and abnormal lipid handling in skeletal muscle. These co-repressors can influence the expression of genes associated with slow-twitch and fast-twitch muscle fibers, potentially affecting muscle contractile properties and performance. Moreover, dysregulation of these co-repressors can effect various performances including; altering the skeletal muscle ability to respond to exercise stimuli and adapt to physical activity, affecting mitochondrial dynamics and oxidative capacity that leads to altered energy production and impaired muscle performance.

## 5 Heart

Previous research has shown that NCoR1 KO increases MEF2D transcriptional activity in skeletal muscle (Yamamoto et al., 2011). More recent studies by Li et al. (2019) confirmed that NCoR1 has a suppressive effect on the size of cardiomyocytes. They showed strong evidence for direct interactions between NCoR1, MEF2, and HDACs. MEF2A and MEF2D are expressed in adulthood and are key transcription factors that interfere with NCoR1 and have a positive effect on cardiomyocyte size (Li et al., 2019). Birth defects, like congenital heart defects (CHD), have been linked to maternal diabetes and NCoR1 contributes to CHD in the children of diabetic mothers (Lin et al., 2018). NCoR1 deficiency led to cardiac hypertrophy under physiological conditions and worsened hypertrophy induced by pressure overload (Li et al., 2019), suggesting NCoR1 may be considered as a stress-responsive and cardioprotective regulator during cardiac hypertrophy (Li et al., 2019). NCoR1 was shown to be highly expressed in the mouse heart and to be significantly downregulated after acute myocardial ischemia-reperfusion (MI/R) injury by activation of STAT1 (Janus Kinase/Signal Transducer and Activator of Transcription) pathway (Qin et al., 2022). Pro-inflammatory cytokines such as interferons can activate the JAK/STAT1 pathway following MI/R damage. Activated, STAT1 forms homo- or heterodimers and translocates to the nucleus, where it binds to GAS (Gamma-activated Sequence) elements. STAT1 may compete with transcription factors that recruit NCoR1 to target gene promoters after it binds to GAS elements. This competition may result in decreased NCoR1 association with its target genes, leading to less co-repression and downregulation of NCoR1-mediated gene silencing. As previously reported (Mottis et al., 2013), NCoR1 typically works in tandem with HDACs to carry out its suppressive functions. HDAC3 enzyme is involved in the repressive activities of NCoR1, and depletion of HDAC3 in cardiomyocytes results in severe cardiac hypertrophy at an early stage (Mottis et al., 2013). It was shown that HDAC3 enzymatic activity has been significantly increased in the hearts of diabetic mice (Xu et al., 2017). RGFP966, a specific HDAC3 enzymatic inhibitor, significantly reduced diabetic cardiomyopathy (DCM), as evidenced by reduced diabetes-induced cardiac dysfunction, hypertrophy, and fibrosis and decreased cardiac oxidative stress, inflammation, and insulin resistance (Xu et al., 2017). It would be interesting to see, if NCoR1 gene silencing could rescue diabetic cardiomyopathy in these mice by inactivating HDAC3 enzymatic activity. Another study found that inhibiting HDAC3 with RGFP966 increased miR-19a-3p and enhanced cardiac function in a rat model of MI/R (Song et al., 2020). In the hearts of rats with streptozotocin (STZ)-induced diabetes, HDAC3 enzymatic activity led to ischemic cardiac damage (Xie et al., 2014; Qiu et al., 2021). Zhang et al. (2018b) reported that HDAC3 overexpression contributed to cardiomyocyte dysfunction. Suppression of HDAC3 enhanced cell survival and decreased apoptosis in a diabetic animal model of cerebral ischemia-reperfusion damage (Zhao et al., 2019). In heart failure (HF) rat model, DNA methyltransferase 1 (DNMT1) expression was increased and HDAC3 deacetylated DNMT1, blocking ubiquitination-mediated degradation (Wang et al., 2022). Heart-specific HDAC3 genetic knockdown significantly reduced the severity of diabetic ischemia-reperfusion damage by reactivating mitophagy via regulation of the Rev-erbα/BMAL1 pathway. HDAC3 induced the expression of FGFs (fibroblast growth factors) and IGFs (insulin-like growth factors), dependent on its deacetylase activity by binding to NCoR1. HDAC3 deletion in epicardium resulted in downregulation of FGFs and IGFs, which are important for development of epicardium-derived cell derivation and migration. More importantly, it was found that in *HDAC3*-deficient epicardial cells, miR-322 and miR-503 were significantly upregulated both *in vitro* and *in vivo*. Inhibition of miR-322 or miR-503 restored the expression of FGF9 and IGF2 in HDAC3 depleted epicardial cells. These findings suggest that HDAC3 physically interact with miR-322/miR-503 and suppresses their expression (Jang et al., 2022). Few studies have also shown that HDAC3 and NCoR1 corepressor complex play an important role in atherosclerosis (Wang et al., 2019; Yu et al., 2020; Huang et al., 2021; Jiang et al., 2022). Circulating monocytes are transported to the subintima during atherosclerosis, where they eventually develop into macrophages. They absorb large amounts of oxidized low-density lipid protein (ox-LDL) to form the foam cells and secrete pro-inflammatory cytokines (like IL-1β, TNF-α, and IFN-γ) that recruit T cells seen in early atherosclerotic lesions (Chen et al., 2018; Yu et al., 2020). Macrophages in mice lacking myeloid HDAC3 switch to an anti-inflammatory response, that produce less inflammatory cytokines, have a lower lipid content in the plaque, and size of the macrophages decreased, indicating a reduction in foam cells formation (Hoeksema et al., 2014). In HDAC3^del^ macrophages, both the PPARγ/LXR pathway and cholesterol efflux were uniformly upregulated (Hoeksema et al., 2014). Mullican et al. showed that the majority of the genes upregulated in HDAC3 knockout macrophages are genes that IL-4 positively regulates in wild-type macrophages. Simultaneously, the genes downregulated are those that IL-4 negatively regulates in wild-type macrophages. This pattern shows that the HDAC3 deletion-induced gene expression program in macrophages is very comparable to the gene expression program associated with alternative activation (Mullican et al., 2011). Overall, it has been shown that HDAC3 and NCoR1 are dysregulated in heart failure and is associated with adverse cardiac remodeling and dysfunction. It influences the expression of genes involved in cardiac hypertrophy, fibrosis, and inflammation, contributing to the maladaptive remodeling of the heart in heart failure. Inhibition of HDAC3 and NCoR1 has been explored as a potential therapeutic strategy to mitigate cardiac remodeling and improve cardiac function in heart failure.

## 6 Inflammation

Inflammation is a key contributor to cardiovascular and metabolic diseases, and their common risk factors comprise high blood pressure, insulin resistance, visceral obesity, and dyslipidemias such as hypercholesterolemia and hypertriglyceridemia. In obesity, activation of chronic inflammation is a fundamental mechanism for reduced insulin sensitivity (Maksymets et al., 2018; Ormazabal et al., 2018). Insulin signaling impairment is caused by the presence of excess pro-inflammatory M1-like macrophages in adipose tissue and the liver (Lumeng et al., 2007), which act on insulin target cells by secreting several cytokines and chemokines. Macrophages in healthy-weight individuals exhibit anti-inflammatory properties, but the polarization of AT macrophages (ATMs) in obese AT changes to a pro-inflammatory phenotype and results in fat accumulation and glucose intolerance (Lumeng et al., 2007; Castoldi et al., 2015; Boulenouar et al., 2017). In obesity, macrophages encircle dead adipocytes (i.e., producing crown-like structures) and produce a variety of pro-inflammatory cytokines that cause local and systemic inflammation and insulin resistance (Lumeng et al., 2007; Haase et al., 2014). Other immune cell types, such as neutrophils, eosinophils and lymphocytes can also contribute to the inflammatory state of the tissue in obesity (Kintscher et al., 2008; Yang et al., 2010), but their primary function in this setting is to regulate macrophage migration and activation. Macrophage inflammatory pathways are tightly regulated by several transcription factors, like NF-κB, adenovirus type 1 (AP1), PPAR family, LXR, and co-activators (NR) and co-repressors (NCoR1/NCoR2) (Glass and Ogawa, 2006).

The most prevalent immune cell population detected in atherosclerotic lesions and plaques is monocyte-derived macrophages. They uncontrollably take up oxidized LDL (oxLDL), release pro-inflammatory cytokines that recruit T cells, and come apart necrotically, increasing plaque development and instability (Moore and Tabas, 2011; Swirski and Nahrendorf, 2013; Geiger et al., 2020). *In vitro* silencing of macrophage NCoR1 results in the same phenotype as activated macrophages: increased production of pro-inflammatory cytokines, chemokines, and metalloproteases, as well as increased macrophage invasiveness (Ghisletti et al., 2009). In the resting state, NCoR1 repress several genes in the inflammatory pathways (Medzhitov and Horng, 2009). NCoR1 dissociates from the promoters upon stimuli and inflammatory pathways are activated, allowing pro-inflammatory transcription factors, such as NF-kB and AP1, to enhance gene expression (Ogawa et al., 2004; Glass and Saijo, 2010). Macrophage-specific deletion of NCoR1 improved insulin sensitivity in obese mice due to an enhanced synthesis of omega (ω) 3 fatty acids (Li et al., 2013). Molecular analyses have shown that the major effect of NCoR1 KO in macrophages is the derepression of LXR, which leads to the expression of *de novo* lipogenesis and fatty acid desaturation genes and the production of local anti-inflammatory ω3 fatty acids, which suppress macrophage inflammatory activation (Li et al., 2013).

Recent work by Oppi et al. demonstrated that the deletion of NCoR1 in myeloid cells significantly exacerbated atherosclerosis in the aortic sinus and thoraco-abdominal aorta of low-density lipoprotein receptor (LDLr) knockout animals. Mechanism-wise, NCoR1 binding to the CD36 promoter is known to block PPARγ-driven CD36 expression. Consequently, peritoneal macrophages of NCoR1-deficient mice displayed higher accumulation of oxLDL and foam cell production driven by CD36 (Oppi et al., 2020). Pro- and anti-inflammatory gene expression was upregulated in NCoR1-deficient macrophages. NCoR1-driven PPARγ suppression is also protective in human plaque formation and susceptibility, according to investigations of multiomics datasets acquired from human plaque specimens (Oppi et al., 2020).

Genetic deletion of NCoR1 in macrophages resulted in decreased infarct size and enhanced cardiac function in mice with experimental myocardial infarction, suggesting a critical role for NCoR1 in the heart (Du et al., 2020). Reduced macrophage proliferation and downregulation of inflammatory transcriptional programs (IL-1, IL-6, AP-1, and NFκB) accounted for this phenotype. Previous research has shown that HDAC3 enzymatic activity is elevated in ischemic heart injury (Xie et al., 2014; Qiu et al., 2021). It would be interesting to determine HDAC3 enzymatic activity in NCoR1 KO macrophages, given that KO of NCoR1 might alter HDAC3 activity. Thus, the protection shown in NCoR1 KO macrophages may also be correlated with HDAC3 enzymatic activity. Consequently, macrophage NCoR1 operates as an upstream regulator of myocardial inflammation, contributing to left ventricular hypertrophy, diastolic dysfunction, and microvascular pathology (Metra and Teerlink, 2017). Neointimal hyperplasia and vascular remodeling were significantly suppressed when NCoR1 was depleted in macrophages in a mouse model of arterial wire damage (Du et al., 2020). These findings imply that deletion of NCoR1 in macrophages may have significant benefits for the prevention of heart failure. Further molecular research and preclinical investigations are necessary to investigate the potential of NCoR-targeting strategies in cardiovascular diseases.

Myeloid cell-specific HDAC3 knockout mice (MHD3KO) have a similar, but not identical, phenotype as compared to a NCoR1-marcophage KO (Oppi et al., 2020). A study by Hoeksema et al. (2014) found that HDAC3-marcophage deficient animals had significantly higher rates of atherosclerotic plaque development in the aortic sinus compared to control mice. In addition, MHD3KO mice exhibited similar behavior to anti-inflammatory to wound healing. In contrast, the atherosclerosis plaques of MHD3KO mice displayed an enhanced collagen distribution and expanded protective fibrous caps, whereas the atherosclerotic plaques of myeloid cell-specific NCoR1 knockout animals exhibited increased necrotic cores (Oppi et al., 2020). HDAC3 deletion attenuated the TLR4-mediated hyperinflammatory response to LPS in macrophages by inhibiting activation of pro-inflammatory genes, yet the mechanism remains elusive (Chen et al., 2012).

**FIGURE 4:**
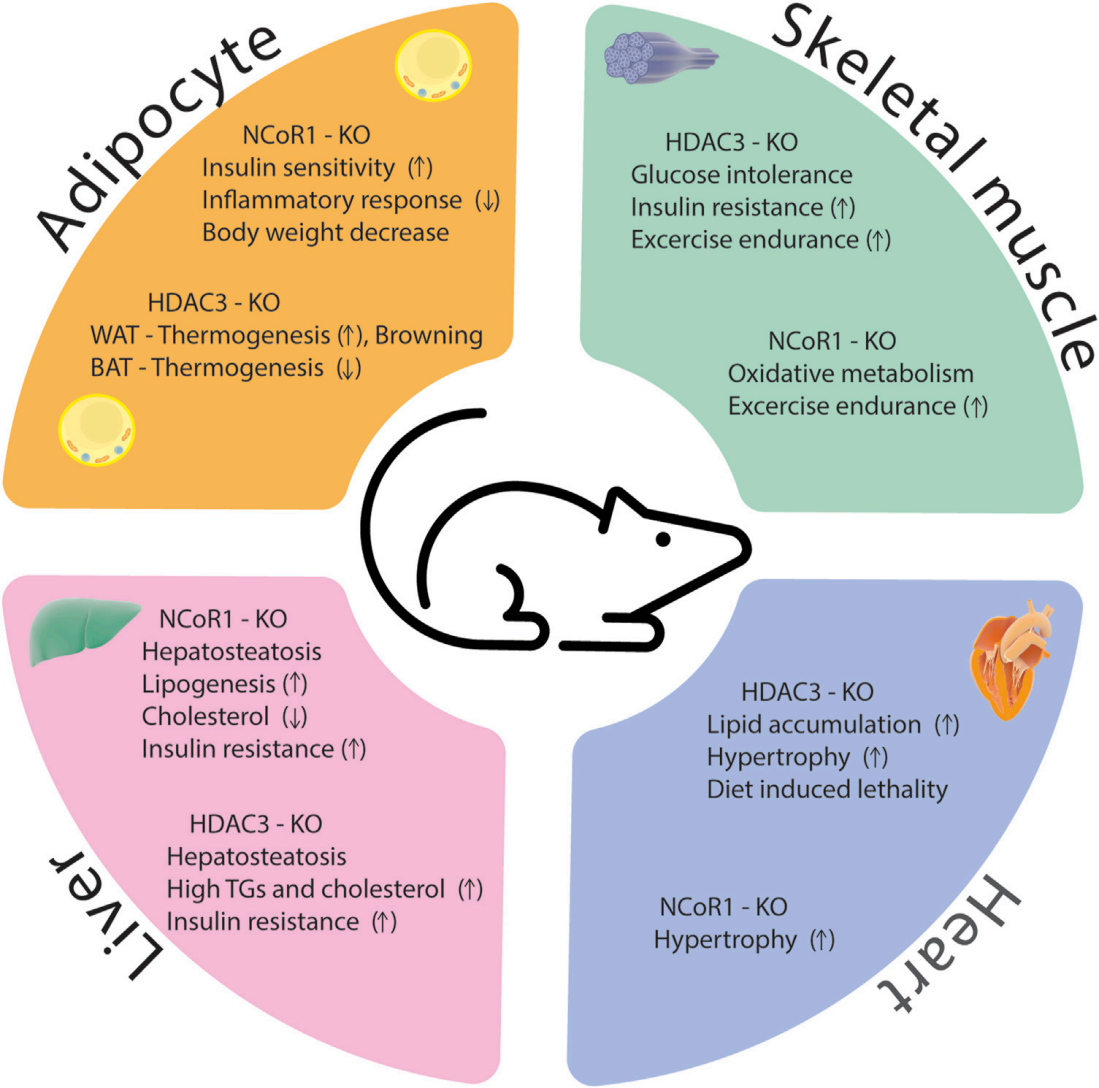
Phenotypes of HDAC3 and NCoR1 knockout models in different metabolic tissues.

The HDAC3/NCoR1 co-repressor complex is believed to serve as a transcriptional gatekeeper for several inflammatory genes. When no nuclear receptor ligand is present, the co-repressor complex is recruited to target genes where it interacts with unphosphorylated c-Jun bound to target promoters, preventing the expression of pro-inflammatory genes. Upon treatment with an innate inflammatory stimulus, such as LPS, the ligand-binding domain of nuclear receptor undergoes a structural change that inhibits its affinity to NCoR1 while simultaneously boosting its affinity to co-activators (Ogawa et al., 2004). Ubiquitin E3 ligase TBLR1 regulates the ligand-dependent ubiquitination and proteasome-mediated degradation of NCoR1 and HDAC3 from the promoters of target genes and results in gene activation (Perissi et al., 2004). Other study showed that B-cell lymphoma 6 (Bcl6) interaction with NCoR1/HDAC3 corepressor complexes is required to facilitate its transrepressive activity in a variety of biological activities (Huynh and Bardwell, 1998; Cardenas et al., 2017). Bcl6 deletion in the liver inhibits PPARα-driven enzymes that mediate fatty acid oxidation, protecting against high-fat diet-induced hepatic steatosis (Sommars et al., 2019). In Bcl6-deficient livers, binding of the corepressors NCoR1, NCoR2, and HDAC3 to BCL6-binding sites was reduced, and these sites demonstrated increased enhancer/promoter activity as measured by increased histone 3 lysine 27 acetylation (H3K27ac) (Sommars et al., 2019). According to these findings, hepatic BCL6 recruits a subset of NCoR/HDAC3 complexes to the promoters of certain target genes that regulate lipid metabolism. In conclusion, despite significant progress in understanding how HDAC3 and NCoR1 control inflammation in metabolic disorders, there are still various areas that researchers can explore to deepen our knowledge and identify potential therapeutic strategies. A comprehensive identification of target genes and pathways regulated by HDAC3 and NCoR1 in the context of inflammation and metabolic diseases is essential for the drug development. Phenotypes of HDAC3 and NCoR1/2 KO models were summarized in Supplementary Table S1 and Figure 4.

## 7 Conclusion

In conclusion, HDAC3, NCoR1, and NCoR2 play a major role in regulating metabolism and have been implicated in several metabolic diseases. Any deficiencies or dysregulations of NCoR1, NCoR2, and HDAC3 have been associated with metabolic diseases such as obesity, type 2 diabetes, and NAFLD. In adipocytes, HDAC3 has been shown to act as a key regulator of energy metabolism and glucose homeostasis. In the liver, NCoR1 and NCoR2 form part of the negative feedback mechanism that controls HDAC3 activity and improve liver function. HDAC3 and NCoR1/NCoR2 have been implicated in regulating insulin sensitivity in skeletal muscle. In the heart, HDAC3 has been shown to protect against cardiac inflammation and metabolic stress. Hence, studying these molecules in greater detail is crucial to understand potential mechanisms underlying metabolic diseases and could contribute to the development of new therapeutic strategies.

## 8 Open questions

1. What are the precise molecular mechanisms causing HDAC3/NCoR1 corepressor complex dysregulation in metabolic diseases such as type 2 diabetes, obesity and cardiovascular disease?
2. How does the HDAC3/NCoR1 corepressor complex interact with other regulatory proteins and signaling pathways involved in metabolic disorders, including as insulin signaling, AMP-activated protein kinase (AMPK) signaling, and the PPAR signaling pathway?
3. Is targeting the HDAC3/NCoR1 corepressor complex an effective therapeutic strategy for metabolic diseases? If this is the case, what are the potential side effects of such treatments?
4. How does the HDAC3/NCoR1 corepressor complex regulate the metabolic switch between oxidative phosphorylation and glycolysis, and what are the implications of this regulation for metabolic disease development?
5. Can dietary treatments, such as calorie restriction or nutrient supplementation, affect the enzymatic activity of the HDAC3/NCoR1 complex to promote metabolic health in mouse model?
6. How does HDAC3/NCoR1 corepressor complex interact with other transcriptional co-regulators, such as HATs and chromatin remodeling complexes, to modulate metabolic gene expression?
