# Supplementary material for: Insights into the function of HDAC3 and NCoR1/NCoR2 co-repressor complex in metabolic diseases
Source: Front Mol Biosci. 2023 Aug 22;10:1190094. doi: 10.3389/fmolb.2023.1190094 (PMC10477789; doi:10.3389/fmolb.2023.1190094)
Supplement: Supplementary file 1 [file Table1.DOCX]

| **Protein name (gene name)** | **Mouse Model** | **Tissue expression** | **Lethality** | **Target TF** | **Key features of the phenotype** | **References** |
| --- | --- | --- | --- | --- | --- | --- |
| **NCOR1** | | | | | | |
| NCoR1 | Knockout | Global | Yes | RAR, T3R | - Embryonic lethality at E 15.5, and developmental defects | (19) |
| NCoR1δRID | Knockin (deletion of RID domains) | Global | No | TR | - Improved insulin resistance and energy expenditure | (52, 135, 136) |
| NCoR1 DADm | Y478A Knockin | Global | No | PGC-1α, TR, LXR | - Abnormal circadian behavior - Improved insulin-sensitivity, and diet-resistant - weight gain | (52) |
| NCoR1δRID | Knockin | Liver | No | TR | - Increased expression of TR - Improved cholesterol tolerance. | (72, 137) |
| NCoR1 | Knockout | Liver | No | LXR, RevErb | - Hepatosteatosis due to increased expression of lipogenesis | (12) |
| NCoR1 | Knockout | Skeletal muscle | No | MEF2, PPARβ/δ, and ERRs | - Increased exercise endurance - Induction of oxidative metabolism | (86) |
| NCoR1 | Knockout | Adipo-cre | No | - | - Increased insulin sensitivity - Decreased inflammatory response - Smaller adipocytes | (46) |
| NCoR1 | Knockout | Heart | No | MEF2A, MEF2D | - Hypertrophy | (91) |
| NCoR1 | Knockout | Macrop-hages | No | CD36, PPARγ | - Activation of pro-inflammatory cytokines | (127) |
| NCoR1 DADm | Y478A Knockin | Global | No | PGC-1α | - Abnormal circadian behavior - Diet-resistant weight gain | (52) |
| **NCoR2** | | | | | | |
| NCoR2 | Knockout | Global | Yes | JMJD3, RAR | - Embryonic lethality at E16.5, lethal heart defect - Impairment of forebrain development | (138) |
| NCoR2 mRID | Knockin | Global | No | TR, PPARγ | - Metabolic defects (insulin resistance, increased adiposity, reduced energy expenditure) | (139) |
| NCoR2 mRID1 | Knockin | Global | No | PPARγ, LXRα | - Lipid accumulation in white and brown adipose tissue - Metabolic dysfunctions | (53) |
| **HDAC3** | | | | | | |
| HDAC3 | Knockout | Global | Yes | H3S10 phosphorylation, DNA damage | - Embryonic lethality due to developmental defects | (27) |
| HDAC3 | Knockout | Adipo-cre | No | Pparγ  and Ucp1 | - Increase of thermogenesis, browning of white adipose tissue | (25) |
| HDAC3 | Knockout | Liver /White adipocytes | No | NCoR, PGC-1α, PPARα/γ, REV-ERBa | - Increase of lipogenesis - Decrease fatty acid oxidation | (12, 25, 30) |
| HDAC3 | Knockout | Brown adipocytes | No | UCP1, PGC-1α, OXPHOS | - Decrease of thermogenesis, weight loss, hypoglycemia | (49) |
| HDAC3 | Knockout | Heart and skeletal muscle | Upon high fat diet | - | - Lipid accumulation - Severe hypertrophic cardiomyopathy | (90) |
| HDAC3 | Knockout | Heart | No | - | - Hypertrophic cardiomyopathy and diet induced lethality | (90) |
| HDAC3 | Knockout | Liver | No | PPARγ | - Hepatosteatosis - Insulin resistance - High TGs and cholesterol | (65) |
| HDAC3 | Knockout | Skeletal muscle | No | AMPD3 | - Gucose intolerance - Muscle insulin resistance - Enhanced exercise capacity | (87) |

TF: Transcription factors
